# Alpha-cell paracrine signaling in the regulation of beta-cell insulin secretion

**DOI:** 10.3389/fendo.2022.934775

**Published:** 2022-07-26

**Authors:** Marlena M. Holter, Mridusmita Saikia, Bethany P. Cummings

**Affiliations:** ^1^ Department of Biomedical Sciences, College of Veterinary Medicine, Cornell University, Ithaca, NY, United States; ^2^ Nancy E. and Peter C. Meinig School of Biomedical Engineering, Cornell University, Ithaca, NY, United States; ^3^ School of Medicine, Department of Surgery, Center for Alimentary and Metabolic Sciences, University of California, Davis, Sacramento, CA, United States

**Keywords:** alpha-cell, beta-cell, paracrine signaling, GLP-1, glucagon, insulin

## Abstract

As an incretin hormone, glucagon-like peptide 1 (GLP-1) lowers blood glucose levels by enhancing glucose-stimulated insulin secretion from pancreatic beta-cells. Therapies targeting the GLP-1 receptor (GLP-1R) use the classical incretin model as a physiological framework in which GLP-1 secreted from enteroendocrine L-cells acts on the beta-cell GLP-1R. However, this model has come into question, as evidence demonstrating local, intra-islet GLP-1 production has advanced the competing hypothesis that the incretin activity of GLP-1 may reflect paracrine signaling of GLP-1 from alpha-cells on GLP-1Rs on beta-cells. Additionally, recent studies suggest that alpha-cell-derived glucagon can serve as an additional, albeit less potent, ligand for the beta-cell GLP-1R, thereby expanding the role of alpha-cells beyond that of a counterregulatory cell type. Efforts to understand the role of the alpha-cell in the regulation of islet function have revealed both transcriptional and functional heterogeneity within the alpha-cell population. Further analysis of this heterogeneity suggests that functionally distinct alpha-cell subpopulations display alterations in islet hormone profile. Thus, the role of the alpha-cell in glucose homeostasis has evolved in recent years, such that alpha-cell to beta-cell communication now presents a critical axis regulating the functional capacity of beta-cells. Herein, we describe and integrate recent advances in our understanding of the impact of alpha-cell paracrine signaling on insulin secretory dynamics and how this intra-islet crosstalk more broadly contributes to whole-body glucose regulation in health and under metabolic stress. Moreover, we explore how these conceptual changes in our understanding of intra-islet GLP-1 biology may impact our understanding of the mechanisms of incretin-based therapeutics.

## Introduction

Pancreatic islet alpha- and beta-cells are traditionally considered to function in opposition to one another, in that alpha-cell-derived glucagon increases hepatic glucose production and beta-cell-derived insulin lowers blood glucose. Failure of precise interplay of alpha- and beta-cell function is central to the pathogenesis of type 1 (T1DM) and type 2 (T2DM) diabetes mellitus. While we typically think of diabetes pathogenesis as mainly being due to a deficiency in beta-cell insulin production, overproduction of glucagon is a significant contributor to diabetes pathogenesis (1–3). The increased appreciation for the role of alpha-cells in the pathogenesis of diabetes has led to more comprehensive investigation of the paracrine signaling networks required for normal islet function. In doing so, recent studies have demonstrated that alpha-cell-derived proglucagon peptides contribute to beta-cell glucose-stimulated insulin secretion (GSIS) (4–9), suggesting that alpha-cell regulation of glucose homeostasis and beta-function is more complex than previously appreciated.

Proglucagon is the precursor to glucagon and glucagon-like peptide-1 (GLP-1). Proglucagon is found in alpha-cells, enteroendocrine L cells in the gut, and in certain neuronal populations. Proglucagon is differentially processed depending on the prohormone convertase (PC) profile of a cell. PC2 (gene: *Pcsk2*) cleaves proglucagon to glucagon, and PC1/3 (gene: *Pcsk1*) cleaves proglucagon to GLP-1 (10, 11). Alpha-cells mostly express PC2; however, an increasing body of literature demonstrates that under certain circumstances, alpha-cells can produce GLP-1 (7, 12–19). GLP-1 is an incretin hormone, meaning that it potentiates beta-cell insulin secretion in a glucose-dependent manner. The canonical model by which GLP-1 has been described to induce its incretin effect is that ingestion of a meal stimulates the release of GLP-1 from gut L cells; this GLP-1 then travels through the blood to the beta-cell GLP-1 receptor (GLP-1R), where it signals to enhance GSIS. In recent years, the field has become increasingly skeptical of this model of GLP-1 incretin function, as circulating levels of active GLP-1 are low, in large part, due to the rapid degradation of active GLP-1 by dipeptidyl-peptidase-IV (DPP-IV) (20, 21). Thus, it is difficult to rationalize how a hormone that contributes to such an important physiological effect is able to do so at such low circulating concentrations. Accordingly, evidence demonstrating the existence of islet GLP-1 production has fostered the alternative hypothesis that the incretin activity of GLP-1 may represent, at least in part, local alpha-cell-derived GLP-1 functioning in a paracrine manner at the beta-cell GLP-1R.

The ability of alpha-cells to switch to GLP-1 as their main endocrine output represents an enormous therapeutic opportunity to manage diabetes. To capitalize on this opportunity, we need to understand the mechanisms that determine alpha-cell GLP-1 production and the physiology of GLP-1 expressing alpha-cells. To this end, our group has found that enhanced beta-cell GLP-1R signaling activates alpha-cell GLP-1 production (13, 16), pointing to an important bi-directional alpha- to beta-cell crosstalk system. This review will explore these and other recent advances in our understanding of alpha- and beta-cell paracrine interactions that dictate islet function and insulin secretion. Additionally, we will investigate how failure of the interplay between alpha- and beta-cell function contributes to the pathophysiology of diabetes, and furthermore, we will highlight how intra-islet paracrine signaling contributes to the effects of pharmaceutical targeting of the GLP-1R.

## Role of the alpha-cell in GSIS

### Proglucagon-derived products signal through the beta-cell GLP-1 receptor and glucagon receptor to potentiate GSIS

**Figure 1:**
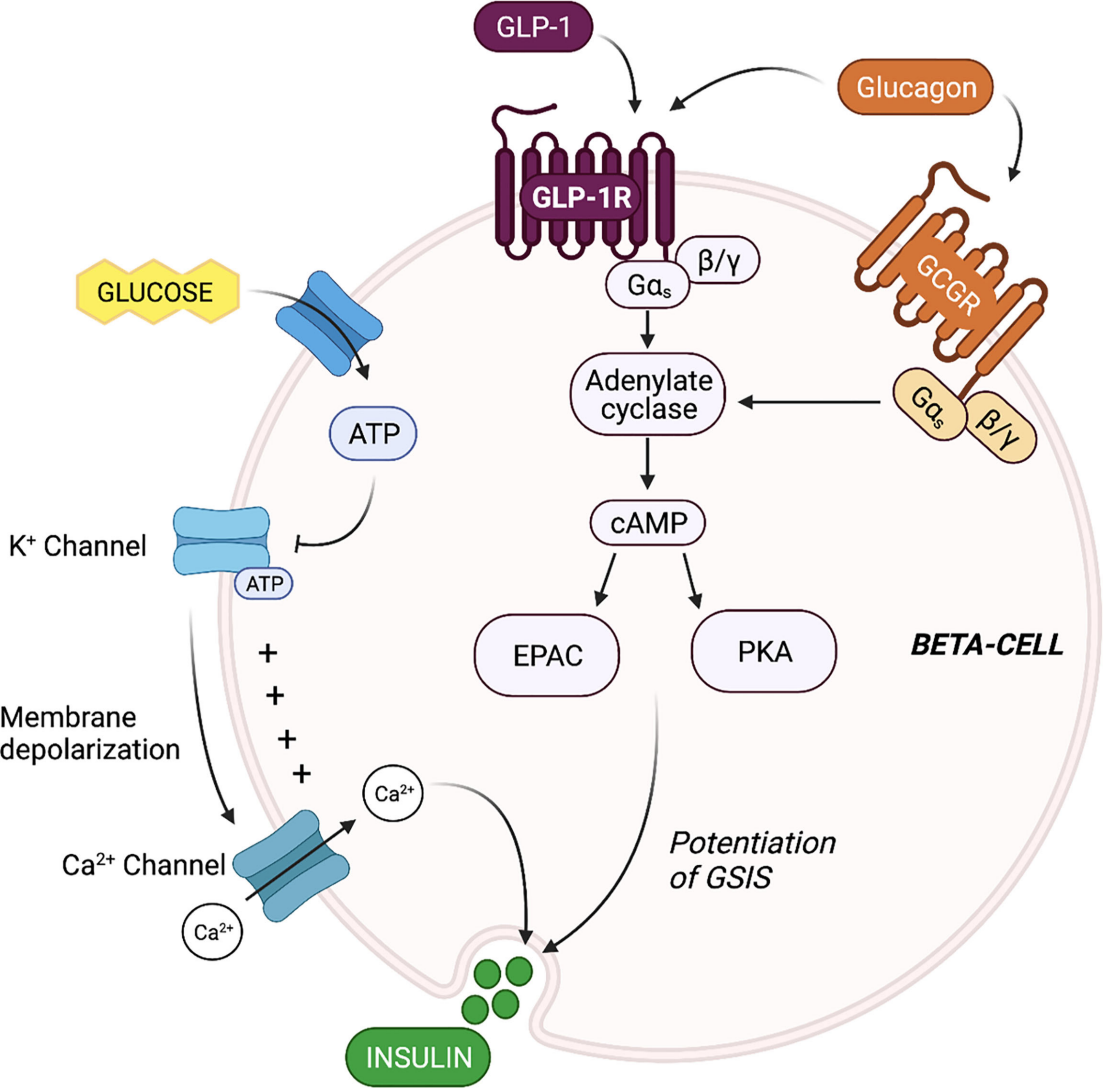
Proglucagon-derived products signal through the beta-cell glucagon-like peptide 1 (GLP-1) receptor and glucagon receptor to potentiate glucose-stimulated insulin secretion (GSIS). The triggering pathway for beta-cell insulin secretion is activated by ATP generation following the influx of glucose. The increase in intracellular ATP induces closure of ATP-dependent K^+^ (K_ATP_) channels, resulting in membrane depolarization and the subsequent opening of the voltage-gated Ca^2+^ channels. The rise in intracellular Ca^2+^ concentrations triggers exocytosis of insulin-containing granules. Stimulation of the beta-cell GLP-1 receptor (GLP-1R) and glucagon receptor (GCGR) results in activation of adenylyl cyclase, leading to increase cAMP production, and the activation of EPAC and protein kinase A (PKA). EPAC and PKA further increase intracellular Ca^2+^ concentrations, allowing for the potentiation of GSIS.

Evaluation of the mechanisms by which beta-cell GLP-1R activation potentiates insulin secretion offers insight into the propensity for alpha-cell-derived proglucagon products to modulate beta-cell function. The triggering pathway for beta-cell GSIS is driven by an increase in ATP and an influx of Ca^2+^. ATP generation following the influx of glucose into the beta-cell induces closure of ATP-dependent K^+^ (K_ATP_) channels, resulting in membrane depolarization and the subsequent opening of the voltage-gated Ca^2+^ channels. This influx of Ca^2+^ then triggers exocytosis of insulin-containing granules. GLP-1 binds to the GLP-1R, which then results in interaction with the G_s_ subunit and activation of adenylyl cyclase. Subsequent increases in intracellular cAMP levels activates PKA and EPAC (22–24), which augment intracellular Ca^2+^ concentrations, allowing for the potentiation of glucose-stimulated insulin granule exocytosis (22, 25). Thus, at the cell signaling level, beta-cell insulin secretion is mediated by glucose metabolism in combination with intra-cellular cAMP levels, which can be potentiated by GLP-1 (**Figure 1**).

Beta-cells also express the glucagon receptor (GCGR), which has significant structural homology and functional overlap with the GLP-1R (26). Several groups have demonstrated that the downstream signaling events of beta-cell glucagon signaling closely align with known signaling cascades of beta-cell GLP-1 signaling (27) (**Figure 1**). For instance, Moens et al. demonstrated the effect of glucagon to stimulate beta-cell cAMP production specifically through the GCGR (28). In addition, Dalle et al. reported that treatment of MIN-6 beta-cells with glucagon resulted in PKA activation and an increase in intra-cellular Ca^2+^ levels, and ERK1/2 activation (29), and furthermore, Jiang et al. reported that the stable expression of the GCGR in human embryonic kidney 293 cells led to similar increases in cAMP and intracellular Ca^2+^ levels in response to glucagon treatment, and dose-dependent activation of ERK1/2 (30), highlighting the GCGR-dependent nature of the effect. These findings indicate that in addition to mediating an increase in cAMP levels, beta-cell glucagon signaling also regulates the phosphorylation of cAMP-response element binding protein (CREB) (29), suggesting that glucagon can directly regulate beta-cell function and survival. Certainly, evidence that glucagon functions as an insulinotropic factor dates back to 1964 when Samols and colleagues performed a glucagon intravenous infusion test and demonstrated that glucagon promotes insulin secretion independent of its effect on blood glucose (31). Ample evidence has since established that the role of glucagon in metabolic regulation extends beyond that of a counterregulatory hormone to include characterization as an insulin secretagogue (6, 8, 28, 32).

Considering the functional and structural similarities between the GCGR and GLP-1R, it was predicted that glucagon mediated these insulinotropic effects through signaling at the beta-cell GCGR. Indeed, Huypens et al. demonstrated that pharmacological inhibition of GCGR activity in dispersed human islets reduced glucagon-induced insulin release (4). However, several studies have since demonstrated that the paracrine regulation of beta-cell function by glucagon can be mediated by the beta-cell GLP-1R, as well (5, 6, 28, 32), in which glucagon can serve as an additional, albeit less potent, ligand for the beta-cell GLP-1R. Moreover, although both GLP-1 and glucagon can bind the GLP-1R, only glucagon can bind the GCGR. However, signaling through both the GLP-1R and the GCGR have been shown to enhance GSIS. Given the overlapping abilities of GLP-1 and glucagon to bind the GLP-1R, the relative contributions of alpha-cell glucagon and GLP-1 to GSIS remain debated.

### Alpha-cell-derived proglucagon products are necessary for normal insulin secretory dynamics

**Figure 2:**
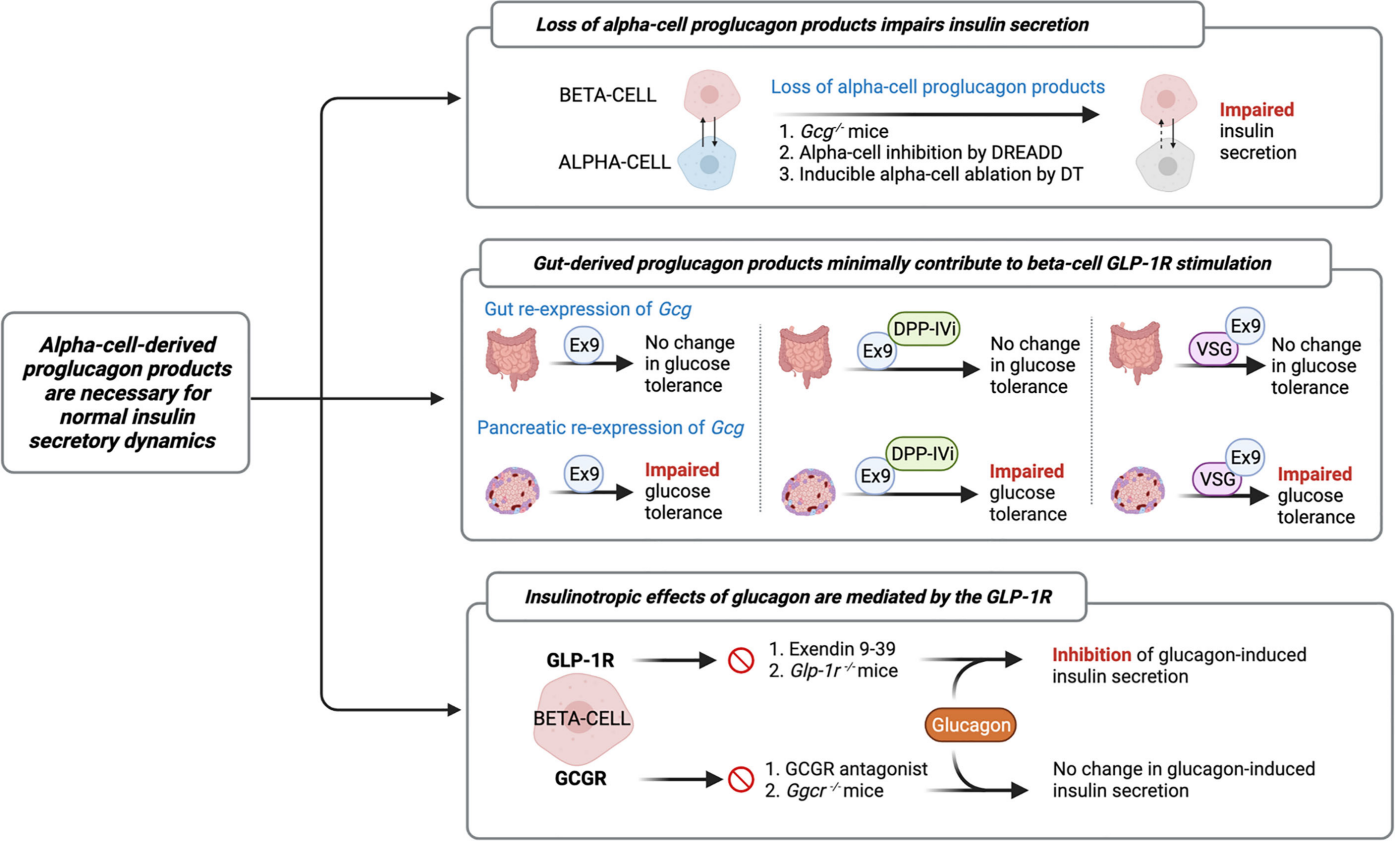
Evidence characterizing the role of the alpha-cell in normal insulin secretory dynamics. Researchers have evaluated insulin secretion from mouse models devoid of all alpha-cell-derived proglucagon peptides (6, 19, 36) and have found that in the absence of paracrine signaling from alpha-cells, beta-cell insulin secretion is impaired. Similarly, several studies have employed mouse models with selective reactivation of *Gcg* expression in the gut or pancreatic islet in order to assess the relative contribution of the gut *vs*. the islet to whole-body glucose tolerance. These studies reveal that islet-derived proglucagon products are the predominant ligand for beta-cell glucagon-like peptide 1 receptor (GLP-1R) activation and are necessary for the glucoregulatory improvements of DPP-IV inhibitors (DPP-IVi) and vertical sleeve gastrectomy (VSG) (15, 37, 38). Furthermore, recent evidence demonstrates that glucagon-induced insulin secretion is primarily mediated by signaling at the GLP-1R. Several independent groups have demonstrated that inhibition of beta-cell GLP-1R signaling, by either Exendin 9-39 (Ex9) or through genetic knockout, impairs glucagon-induced insulin secretion, whereas pharmacological or genetic inhibition of glucagon receptor (GCGR) signaling fails to abolish this effect (5, 6, 8, 28, 36).

The increasing recognition of discrepancies in the canonical model describing the incretin function of GLP-1, along with the identification of islet GLP-1 production, has led the field to study the role of alpha-cell-derived proglucagon peptides in the regulation of beta-cell function. Indeed, early studies by Pipeleers et al. (33) and Holz et al. (34), and more recent studies by Wojtusciszy et al. (35), reported that insulin secretion from isolated beta-cells was blunted as compared to insulin secretion from paired alpha- and beta-cells, providing the initial rationale for alpha-cell contributions to insulin secretion. Several groups have since evaluated insulin secretion from mouse models devoid of all alpha-cell-derived proglucagon peptides (**Figure 2**). For example, Capozzi et al. found that islets from *Gcg^−/−^* mice exhibit impaired GSIS, which could be rescued by exogenous stimulation with glucagon and/or GLP-1 (6). Similarly, Zhu et al. (36), using a DREADD mouse model, and Traub et al. (19), using a mouse model with inducible alpha-cell ablation, both reported that acute loss of alpha-cell secretory products results in impaired insulin secretion. The combined observations from these various mouse models reveal an important role for the alpha-cell in the support of healthy beta-cell function.

In further characterizing this paracrine effect, the field has employed several different models of selective inhibition of proglucagon expression to more directly investigate the relative contributions of gut- versus islet-derived proglucagon products to glucose homeostasis (**Figure 2**). For example, Chambers et al. reported that *Gcg^−/−^* mice with selective re-expression of intestinal proglucagon did not exhibit glucose intolerance in response to treatment with the GLP-1R antagonist, Exendin 9-39, whereas mice with selective re-expression of pancreatic proglucagon did exhibit glucose intolerance in response to Exendin 9-39 (37). Using a similar approach, Hutch et al. demonstrated that pancreatic, but not intestinal, proglucagon-derived peptides are necessary for the glucoregulatory improvements of DPP-IV inhibitors (15), thereby providing further support for the concept that islet-derived GLP-1 contributes to glucose regulation. Furthermore, in *Gcg^−/−^* mice with selective re-expression of *Gcg* in the pancreas or the gut revealed a role for pancreatic, but not intestinal, proglucagon products, in the metabolic benefits of vertical sleeve gastrectomy (VSG) (38). These findings are consistent with our work in which beta-cell GLP-1R signaling was found to contribute to improved islet function after VSG (39), which was associated with a VSG-induced beta-cell GLP-1R-dependent increase in islet GLP-1 production in mice (16). In comparison, Song et al. utilized mouse models with selective elimination of proglucagon expression in the distal gut versus the entire gut and found that gut-derived GLP-1 is the predominant source of increased circulating GLP-1 following nutrient ingestion, slows gut motility, and contributes to maintenance of oral glucose tolerance, but does not contribute to insulin secretion (40). Overall, while these independent studies suggest that gut-derived GLP-1 minimally contributes to beta-cell GLP-1R stimulation, a fundamental limitation to these interpretations is that these studies do not specifically manipulate alpha-cell GLP-1, as these models are deficient in all proglucagon-derived peptides.

Indeed, this limitation is increasingly relevant in light of evidence that the insulinotropic effects of glucagon are predominantly mediated by the GLP-1R (**Figure 2**). In an early study, Moens et al. reported that glucagon-induced insulin secretion was inhibited by Exendin 9-39 in purified rat beta-cells (28). Similarly, treatment of perfused mouse islets with Exendin 9-39 decreased GSIS, but Ado, a selective glucagon receptor antagonist, had no effect on GSIS (36), demonstrating that the effect of alpha-cell-derived proglucagon products to increase insulin secretion does not necessitate signaling through the GCGR. Consistent with this observation, Capozzi et al. reported that neither the selective deletion of beta-cell GCGR signaling nor incubation with a glucagon receptor antagonist, abolished glucagon-induced insulin secretion from isolated mouse islets (6). In contrast, this same group found that selective deletion of the beta-cell GLP-1R and treatment with Exendin 9-39 reduced glucagon-stimulated insulin secretion (6). Similarly, Svendsen et al. found that the combination of GCGR knockout and Exendin 9-39 in perfused mouse pancreata completely abolished glucagon-induced insulin release, whereas the independent genetic ablation of GCGR or GLP-1R had no effect (5). More recently, Zhang et al. reported that under basal glucose conditions, GSIS signals through both the GLP-1R and the GCGR; however, under high glucose conditions, glucagon primarily signals through the GLP-1R (8), suggesting that the paracrine actions of glucagon signaling are influenced by metabolic state. Together, these results demonstrate that while the insulinotropic effects of glucagon are primarily mediated by signaling through the GLP-1R, beta-cell GCGR signaling contributes, at least in part, to the effect.

Nevertheless, the propensity for glucagon to augment insulin secretion through the beta-cell GLP-1R adds another layer of complexity to characterizing the physiological importance of alpha-cell-derived GLP-1 to the incretin effect. While GLP-1 is 300-fold more potent at stimulating beta-cell GLP-1R-induced GSIS than glucagon (6), glucagon is produced at much higher levels in healthy islets (41, 42). Further work is needed to identify the primary ligand for the beta-cell GLP-1R in health and disease. Nevertheless, the present studies establish that intra-islet ligands for the beta-cell GLP-1R are key contributors to normal insulin secretion, supporting the hypothesis that alpha-cell proglucagon-derived products contribute to GSIS.

## Alpha-cell GLP-1 production

### Evidence of intra-islet GLP-1 production

In recent years, data from multiple groups have documented the existence of islet GLP-1 production. The potential for islet GLP-1 production stems from the processing of its precursor molecule, proglucagon. GLP-1 is encoded by preproglucagon (gene name, *Gcg*) and is expressed in neurons in the nucleus tractus solitarus, enteroendocrine L cells, and pancreatic alpha-cells (43–45). Enzymatic post-translational processing of proglucagon renders various peptide hormones depending on the relative expression of prohormone convertase enzymes, PC1/3 and PC2. Classically, in enteroendocrine L cells, proglucagon is cleaved by PC1/3 to produce GLP-1, whereas in the alpha-cell, proglucagon is processed by PC2 to yield glucagon (10, 11). It was previously thought that the tissue-specific processing of proglucagon was due to differential expression of PC1/3 and PC2; however, ample evidence from the field has now demonstrated that alpha-cells can express PC1/3 and produce GLP-1 under certain circumstances (7, 12–16).

Indeed, several early studies using GLP-1 immunoreactivity (46, 47) and HPLC (41, 42, 48) of pancreatic and intestinal tissue sections detected GLP-1 in the pancreatic tissue, albeit at a considerably lower concentration than that of glucagon. However, excitement about pancreatic GLP-1 production was somewhat muted by early work in the perfused pancreas of humans (49) and rats (50) that detected mainly the inactive form of GLP-1 [GLP-1_(1–37)_] and, therefore, called into question the physiological relevance of pancreatic GLP-1. However, using improved detection methods, active GLP-1 has since been identified in isolated human and rodent islet protein lysates (13, 51–55) and in the conditioned media of immortalized alpha-cells (51, 56) and intact primary rodent and human islets (51, 57). Despite these advances, subsequent characterization of the functionality of pancreatic GLP-1 has been more challenging. Several groups have utilized GLP-1R functional endpoints as an indirect indication of the bioactivity of GLP-1 released from isolated islets. For example, Marchetti et al. elegantly demonstrated that islets incubated in media containing human islet supernatants exhibit increased insulin secretion in a GLP-1R-dependent manner (58). Although researchers initially concluded that the GLP-1R-dependent nature of this effect confirmed the bioactivity of islet-derived GLP-1 (58), recent evidence that glucagon can stimulate the GLP-1R complicates this interpretation. Nevertheless, the results of this seminal study confirmed the existence of a functional intra-islet GLP-1R signaling axis in human islets. Consistent with these findings, Hansen et al. reported on the effect of conditioned media from *Psammomys obesus* islets to enhance cAMP production to a similar degree as synthetic GLP-1 in a GLP-1R-expressing cell line (59), thereby providing further evidence that islets secrete functional GLP-1R ligands. In their attempt to delineate the relative contribution of GLP-1 versus glucagon to this effect, Hansen et al. reported that the ability of islet conditioned media to increase cAMP levels was maintained when a high-affinity glucagon antibody was added to the conditioned media, implying that islet-derived GLP-1, rather than glucagon, was the primary active ligand for the GLP-1R in this model. While the regulation of alpha-cell GLP-1 production remains poorly understood, several studies have demonstrated that experimentally induced islet stress (e.g., by a cytokine cocktail (60), streptozotocin (STZ) (51), palmitate (61), or chronic hyperglycemia (18, 59)) potently stimulates alpha-cell GLP-1 production. Such findings suggest that alpha-cells may activate GLP-1 production as a compensatory mechanism to protect beta-cell mass and function. Overall, the combined observations of these early studies, together with more recent evidence, suggest that alpha-cells are capable of processing and secreting active GLP-1.

### Role of islet GLP-1 in alpha-cell heterogeneity

**Figure 3:**
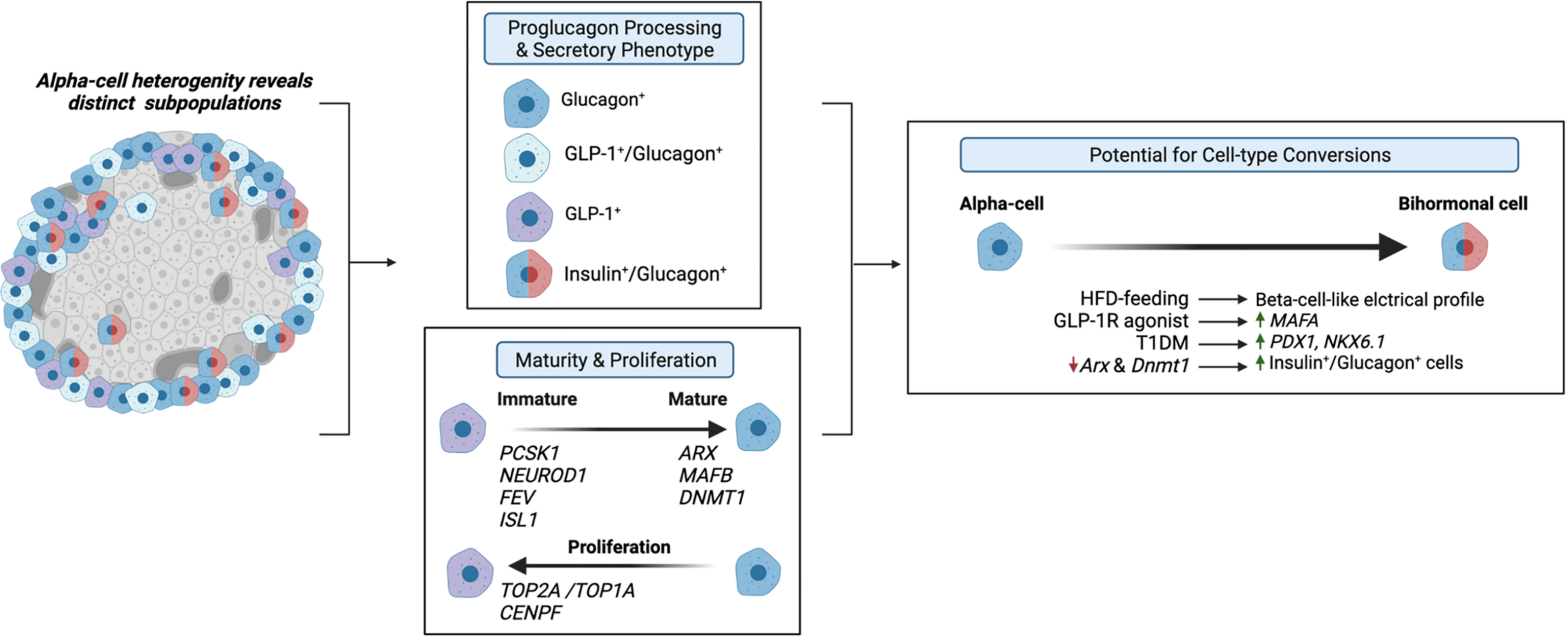
Alpha-cell glucagon-like peptide 1 (GLP-1) serves as a marker for alpha-cell heterogeneity. Several studies have identified distinct alpha-cell subpopulations that differ based on proglucagon processing (7, 12, 62) and secretory phenotype (13, 66, 67), which suggests that these distinct subpopulations can differentially regulate intra-islet paracrine signaling interactions. Similarly, recent evidence suggests that alpha-cell GLP-1 production may reflect an immature alpha-cell, as these cells have been shown to exhibit increased expression of *PCSK1* (13), and increased expression of progenitor markers, *NEUROD1*, *FEV*, and *ISL1* (68, 69) and decreased expression of alpha-cell maturity markers, such as *ARX* and *DNMT1* (13, 70). Additionally, independent studies have identified a subpopulation of proliferating alpha-cells expressing *TOP2A/TOP1A* and *CENPF* (71, 72), which may be associated with maturity state and GLP-1 production. Together, these studies suggest that the heterogeneous proliferative state, maturity, and secretory phenotypes of alpha-cells prime various subpopulations for cell-type conversions. Several studies have demonstrated that under certain conditions, such as high-fat-diet (HFD) feeding (73), GLP-1R agonist treatment (13), T1DM (67), and decreased expression of alpha-cell maturity markers (67) of alpha-cells exhibit beta-cell-like characteristics.

Evidence that the alpha-cell proglucagon processing profile is dependent on the differential expression of PC1/3 versus PC2 suggests that alpha-cell GLP-1 production may function as a marker of alpha-cell heterogeneity. Moreover, given that GLP-1 is a more potent inducer of the beta-cell GLP-1R than glucagon, alpha-cell transcriptional and functional heterogeneity, in terms of GLP-1 production, may importantly affect intra-islet paracrine interactions. To this effect, Zadeh et al. utilized flow cytometry and a fluorescent zinc granule indicator to document the existence of at least two subpopulations of alpha-cells within non-diabetic human islets that differ with respect to their glucagon granule abundances (62). These findings suggest that previously documented alpha-cell heterogeneity within the exocytic capacity (63) and Ca^2+^ responses to glucose (64, 65) may extend to proglucagon processing. In support of this hypothesis, Campbell et al. found that non-diabetic human islets consist of three distinct populations of alpha-cells that differ based on proglucagon-derived peptides: one population that produces GLP-1, one that produces glucagon, and one that produces both GLP-1 and glucagon (12). Moreover, de Souza et al. found that 50% of alpha-cells in healthy mouse islets produce both GLP-1 and glucagon, as compared to 70% of alpha-cells from human islets (7). These studies suggest that differential proglucagon processing between alpha-cells imparts enough dissimilarities between cells to characterize these cells as distinct subpopulations of alpha-cells, and therefore, these studies underscore the possibility that alterations in the proportions of alpha-cell subpopulations that produce either glucagon or GLP-1 or both hormones can have important effects on islet function (**Figure 3**).

Similarly, researchers have proposed that the primary products of alpha-cell proglucagon processing are reflective of alpha-cell maturity state (**Figure 3**). Wilson et al. reported that embryonic glucagon-expressing cells also expressed PC1/3 and that the level of PC1/3 expression decreased with age (70), suggesting that alpha-cell GLP-1 production may be indicative of immature alpha-cells. Thus, considering evidence documenting the existence of various GLP-1-producing subpopulations of alpha-cells in the adult pancreas, these results would indicate that a range of alpha-cell maturity exists within the adult pancreas. Indeed, our single-cell RNA-seq (scRNA-seq) study in human islets found that transcripts that regulate alpha-cell maturity, such as *ARX* and *DNMT1*, are differentially expressed across alpha-cell subpopulations, in which pseudotime analysis suggested that certain subpopulations are more mature than others (13). Moreover, supporting the conclusions of Wilson et al. (70), we found that the most immature subpopulation of alpha-cells also exhibited the highest expression of *PCSK1* (gene name for PC1/3) (13). Additionally, we reported an increase in this immature-*PCSK1*-expressing alpha-cell subpopulation in response to intra-islet GLP-1R signaling (13), suggesting that maturity state and GLP-1 production may be related. Furthermore, increased islet GLP-1 production has been documented in mice with alpha-cell hyperplasia. For example, genetic and pharmaceutical ablation of GCGR signaling potently promotes alpha-cell hyperplasia, which is associated with an increase in alpha-cell GLP-1 expression (5, 74–78). Recently, several single-cell transcriptomic data sets have identified subpopulations of proliferating alpha-cells. In their study performed on both non-diabetic and T2DM human islets, Segerstolpe et al. observed two distinct populations of alpha-cells with similar proglucagon expression but differential expression of proliferation-associated genes (e.g., *TOP2A*, *MKI67*, *CENPF*, *BIRC5*, and *CDK1*), suggesting that one of these subpopulations, albeit the smaller subpopulation, of alpha-cells may constitute proliferating alpha-cells (71). A subsequent scRNA-seq study on human islets documented a similar alpha-cell subpopulation consisting of a small number of proliferating alpha-cells, expressing *TOPA1*, *CENPF*, and *AURKB* (72). Although whether alpha-cell GLP-1 production and maturity state directly relate to alpha-cell proliferative state has yet to be established, these studies identify a range of alpha-cell maturity and proliferative capacity within islets and allude to the possibility that alpha-cell GLP-1 production may be inherently involved in this heterogeneity.

Further technical advances have enabled researchers to validate single-cell transcriptomes with direct functional correlates in the same cell, resulting in more precise measures of alpha-cell heterogeneity. For example, studies using whole-cell patch clamp combined with scRNA-seq analysis indicate functional and transcriptomic heterogeneity in non-diabetic (68) and diabetic (73) human alpha-cells. Notably, a subset of alpha-cells showed enrichment of alpha-cell maturity markers (*LOXL4*, *MAFB*, and *ARX*), regulators of reduced ER stress (*DDIT3*, *XBP1*, and *PPP1R15A*), transcription factors governing endocrine fate (*FEV* and *ISL1*), receptors involved in glucose homeostasis (*FFAR1* and *GPAR119*), and secretory pathways (*SCG2*) (68). Moreover, this transcriptional heterogeneity correlated with electrophysiological and morphological features, including Na^+^ currents and cell size (68). In comparison, Dai et al. recently identified a subpopulation of immature alpha-cells from T2DM human islets that exhibited increased expression of progenitor markers, *NEUROD1*, *FEV*, *ISL1*, and *GATA6*, in addition to *ARX*; however, this group reported that alpha-cells with this immature transcriptional profile also exhibited an impaired electrophysiological phenotype and dysregulated exocytosis (73). These findings not only establish that the alpha-cell transcriptional heterogeneity translates into subpopulations that are functionally distinct but also suggest that specific alpha-cell subpopulations may differentially respond to and contribute to metabolic dysfunction.

In addition to these findings that specific alpha-cell subtypes may contribute to disease progression, recent evidence has fostered the similar hypothesis that the heterogenous proliferative state, maturity, and secretory phenotypes of alpha-cells primes various subpopulations for cell-type conversions (**Figure 3**). To this end, high-fat-diet (HFD) feeding in mice has been shown to induce beta-cell-like changes in the electrical profile of a subset of alpha-cells (73). Moreover, we recently found that alpha-cell subpopulations with decreased expression of alpha-cell maturity markers, *ARX* and *DNMT1*, exhibit increased expression of markers of beta-cell fate, such as *MAFA*, and furthermore, we find this effect in induced in human islets in response to treatment with a GLP-1R agonist (13). These data are consistent with previous works demonstrating that increased GLP-1R signaling promotes alpha-cell transdifferentiation in mice (79, 80). Moreover, our findings parallel that of the work of Chakravarthy et al. in which reductions in *DNMT1* and *ARX* expression in alpha-cells from islets of T1DM donors were associated with increased expression of beta-cell markers, *PDX1* and *NKX6*.*1* (67), and furthermore, this group found that the selective loss of alpha-cell *Arx* and *Dnmt1* expression led to an increase in alpha-cell-derived bihormonal insulin^+^/glucagon^+^ cells in mouse islets (67). Similarly, the findings of Collombat et al. (81) and Thorel et al. (82) provide tangible evidence that alpha-cells can be converted to functional beta-cells in response to beta-cell dysfunction. Although the results of these studies may be promising, future lineage tracing studies are warranted to determine the cellular origins and phenotypes of GLP-1-expressing islet cells. Overall, with several studies investigating alpha-cell heterogeneity, it is tempting to speculate that certain subpopulations of alpha-cells can be harnessed to develop treatment strategies for diabetes.

## Role of alpha-cell GLP-1 production in metabolic dysfunction

### Alpha-cell GLP-1 production as an adaptive response to metabolic dysfunction

**Figure 4:**
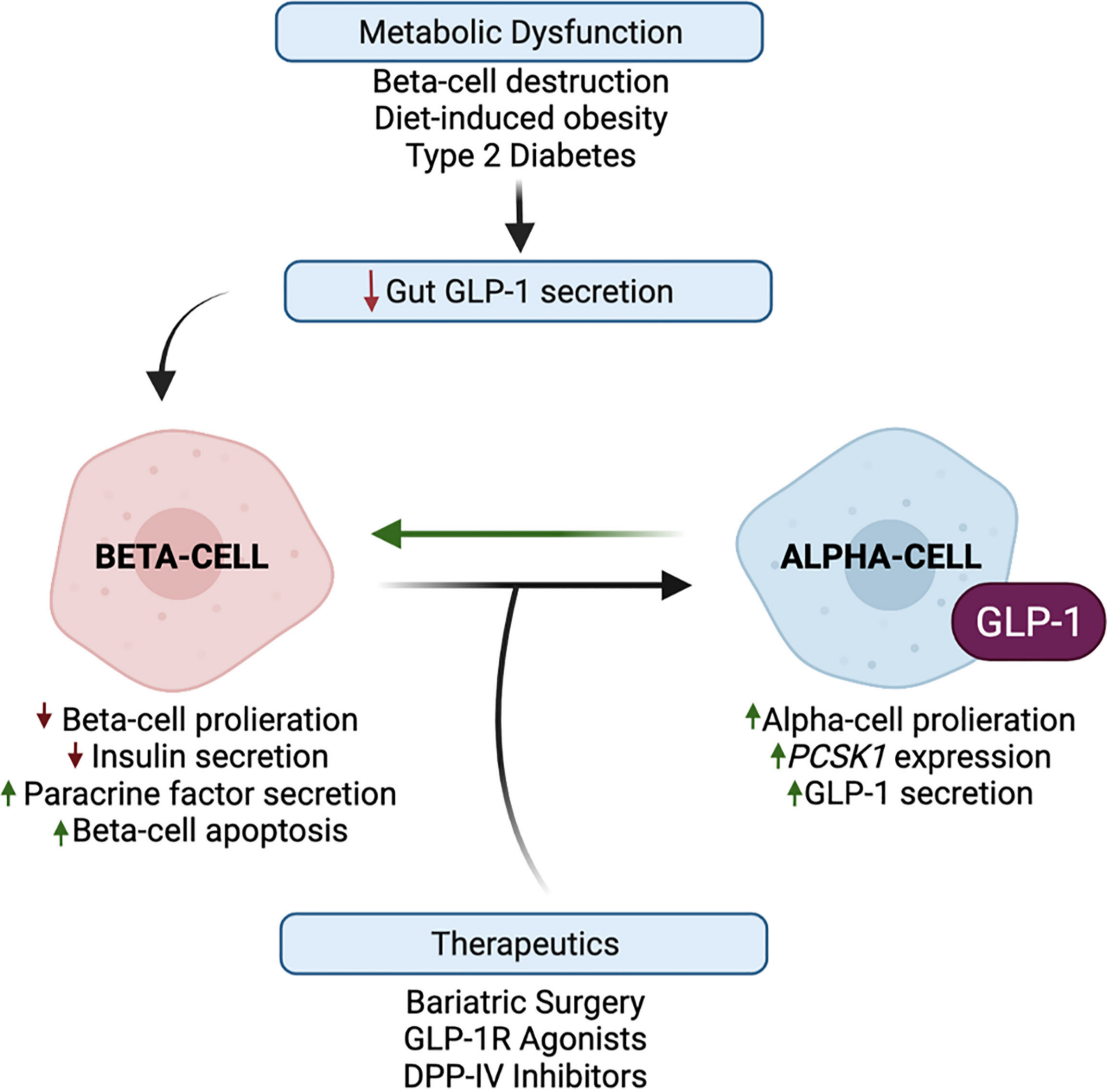
Alpha-cell glucagon-like peptide 1 (GLP-1) production is upregulated in response to metabolic stress and therapeutic interventions. Metabolic dysfunction, mediated by beta-cell destruction, diet-induced obesity, and type 2 diabetes, results in a blunted incretin effect of GLP-1. Evidence suggests that the paracrine action of alpha-cell GLP-1 may compensate for this diminished peripheral incretin action of gut-derived GLP-1. In response to metabolic dysfunction, beta-cells exhibit decreased proliferation and increased apoptosis, and decreased insulin secretion and increased paracrine signaling factor secretion. In response to beta-cell paracrine signaling factors, and whole-body metabolic inputs, alpha-cells exhibit increased proliferation, *PCSK1* expression, and GLP-1 production, which may reduce alpha-cell glucagon secretion. These favorable alpha-cell adaptations can then reciprocally promote beta-cell function, survival, and insulin secretion. Several studies have demonstrated that bariatric surgery and the pharmaceutical targeting of GLP-1 through GLP-1R agonists and DPP-IV inhibitors have the potential to harness and promote alpha-cell GLP-1 production.

Although the *in vivo* physiological function of intra-islet GLP-1 has been questioned since levels of GLP-1 in the pancreas of healthy mice are considerably low and sometimes undetected (5, 83), metabolic stress has been reported to potently increase alpha-cell-derived GLP-1 and decrease L-cell-derived GLP-1 in rodents and humans (18, 58, 84–90). Certainly, human patients with T2DM display a blunted incretin effect of GLP-1, and defects in meal-induced intestinal GLP-1 secretion (91), suggesting that the paracrine action of alpha-cell-derived GLP-1 may be necessary to combat this diminished peripheral incretin action of gut-derived GLP-1. To this effect, the field has generated compelling evidence that beta-cell GLP-1R signaling, mediated by alpha-cell-derived proglucagon products, is an essential component of the adaptation to metabolic stress (**Figure 4**). For example, lean chow-fed *Glp1r ^βcell–/–^:Gcgr^βcell–/–^* mice exhibit normal oral and IP glucose tolerance as compared to wild-type controls; however, metabolically stressed *Glp1r^βcell–/–^:Gcgr^βcell–/–^* mice exhibit impaired oral and IP glucose intolerance as compared to HFD-fed controls (6). In elaboration of these observations, work in an alpha-cell-specific PC1/3 knockout mouse model demonstrates that the loss of alpha-cell GLP-1 production impairs glucose homeostasis and GSIS in metabolically stressed mice but not in chow-fed mice (19). In further support of this adaptive response, islets from T2DM human donors display an increased subpopulation of GLP-1^+^ alpha-cells, and a greater dependency on GLP-1R signaling for insulin secretion, as compared to islets from healthy donors (12). Similarly, O’Malley et al. reported an increase in alpha-cell PC1/3 expression and an increase in the ratio of GLP-1^+^:glucagon^+^ alpha-cells with the onset of diabetes in *db/db* mice as compared to normal mice (86). Moreover, studies performed in sand-rat gerbils, *Psammomys obesus*, a rodent model of nutritionally induced diabetes, document an increase in islet GLP-1 production in response to the development of symptoms of T2DM (59). Together, these results suggest that enhanced alpha-cell GLP-1 production may be an early response to metabolic dysregulation, suggesting that the adaptation to metabolic stress involves a greater reliance on this intra-islet paracrine signaling for glucose homeostasis.

Considering that chronic beta-cell GLP-1R signaling activates cAMP-responsive element binding (CREB) and epidermal growth factor receptor (EGFR) pathways that induce pro-survival and anti-apoptotic responses (92–94), in addition to the acute potentiation of GSIS, the activation of alpha-cell GLP-1 production may also serve to promote the survival and proliferation of the remaining beta-cells (**Figure 4**). Indeed, Wideman et al. explored the beta-cell protective capacity of this increase in alpha-cell GLP-1 production and found that the xenotransplant of PC1/3-expressing alpha-cells into STZ-treated mice improved glucose tolerance and preserved beta-cell mass in a GLP-1R-dependent manner (56). Similarly, STZ-induced alpha-cell GLP-1 production partially protected rat islets from further beta-cell loss (18), demonstrating the functionality of locally produced GLP-1 in long-term beta-cell adaptation. In highlighting the paracrine importance of alpha-cell GLP-1 on beta-cell function, Liu et al. reported that alpha-cells exhibit increased PC1/3 expression and GLP-1 secretion in response to incubation with a chemokine, stromal cell-derived factor-1 (SDF-1), secreted by experimentally injured beta-cells (95); moreover, researchers concluded that reciprocal paracrine signaling by GLP-1, in combination with autocrine signaling by SDF-1, synergistically functions to preserve and enhance beta-cell mass (95). In addition to these protective effects, researchers have also examined the restorative capacity of exogenous GLP-1 in response to beta-cell destruction. Short-term GLP-1 treatment has been shown to enhance recovery from STZ-mediated beta-cell destruction in neonatal rats (96). Similarly, Ogawa et al. reported that treatment of diabetic NOD mice with a combination of immunosuppressive therapy and Exendin-4, a GLP-1 mimetic, resulted in an increase in pancreatic insulin content and complete remission in 90% of treated mice, as compared to mice treated with the independent therapies (97). Given that both T1DM and T2DM are associated with reduced beta-cell mass and function, these various studies highlight the therapeutic potential of harnessing alpha-cell GLP-1 production to not only stimulate insulin secretion but also promote beta-cell survival and regeneration.

Furthermore, as dysregulated glucagon secretion is a key pathogenic mediator of T1DM and T2DM (3), it is necessary to investigate how the activation of alpha-cell GLP-1 production contributes to alpha-cell function. While it was initially proposed that hyperglucagonemia in diabetes results from a loss of insulin inhibitory control on alpha-cell glucagon secretion, an alternative hypothesis is that alpha-cell number and secretory capacity is increased as a compensatory mechanism to enhance beta-cell function. Indeed, patients with T1DM (98) and mouse models of beta-cell destruction (99) report that insulin deficiency is accompanied by an increase in alpha-cell mass, which has served as rationale for the notion that a loss of beta-cell paracrine signaling drives alpha-cell proliferation. However, HFD-feeding has been shown to induce alpha-cell hyperplasia prior to the expansion of beta-cell mass in mice (17, 100), and similarly, studies performed in normoglycemic non-human primates demonstrate that alpha-cell mass was more significantly augmented in response to the increased duration and severity of obesity than that of beta-cells (101). Together, these results reveal that obesity-induced changes in beta-cell function may parallel or follow, rather than precede, increases in alpha-cell number, suggesting that alpha-cell proliferation may serve as a pro-active, as opposed to reactive, response to metabolic dysfunction (**Figure 4**).

Alpha-cell maturity and proliferative state is also associated with GLP-1 production. Metabolic conditions that increase alpha-cell mass (9, 17, 44) also increase alpha-cell PC1/3 expression (13, 61) and GLP-1 production (41, 75, 86). More direct evidence of this effect was identified by Vasu et al., which reported that alpha-cell proliferation in response to STZ-treatment was specifically associated with an increase in GLP-1^+^ alpha-cells (102). Similarly, Whalley et al. reported that STZ-treated rat islets exhibit increased GLP-1 secretion with no changes in glucagon secretion (51). However, increased plasma glucagon concentrations have been reported in mice with HFD-induced increases in alpha-cell mass (9), indicating that alpha-cell proliferation does not solely increase GLP-1 secretion. Since it is unknown whether increasing alpha-cell GLP-1 occurs at the expense of glucagon, these conflicting observations raise important questions as to whether compensatory alpha-cell proliferation during the development of insulin resistance or insulin insufficiency exacerbates or mitigates glucagon-induced hyperglycemia. Certainly, independent groups have demonstrated that mice with marked alpha-cell hyperplasia and hyperglucagonemia secondary to whole-body GCGR knockout are resistant to the metabolic manifestations of beta-cell destruction (103, 104); however, evidence suggests that this resistance is mediated by compensatory increases in whole-body GLP-1R signaling (105), and increased islet GLP-1 production (18), indicating that a GLP-1R-mediated mechanism has a protective effect on glucose homeostasis in mice with alpha-cell proliferation. These results suggest that adjunctive therapies that inhibit glucagon peripheral actions and activate local GLP-1 signaling may improve diabetes treatment (105, 106). To this end, further work is needed to address whether increasing alpha-cell PC1/3 expression can shift alpha-cell proglucagon processing to favor GLP-1 production over glucagon, thereby mitigating the metabolic consequences of hyperglucagonemia. If so, defining the molecular trigger(s) that regulates alpha-cell PC1/3 expression has the potential to identify targeting strategies that can switch alpha-cells from being diabetes promoting to diabetes preventing.

### Role of intra-islet paracrine signaling in the effects of pharmaceutical targeting of GLP-1

As current therapies focusing on increasing insulin sensitivity and/or insulin secretion fail to cure diabetes, one potentially underappreciated target for diabetes treatment is the alpha-cell. Considering the bihormonal nature of the pathophysiology of diabetes, the glucose-dependent insulinotropic and glucagonostatic properties of GLP-1 have made GLP-1 an ideal candidate for the treatment of T1DM and T2DM. However, as these drugs utilize the classical incretin concept as a physiologicy framework, we are challenged to reassess the mechanism of action of these drugs in light of our refined understanding of GLP-1 incretin function and alpha-cell regulation of beta-cell function.

**Figure 5:**
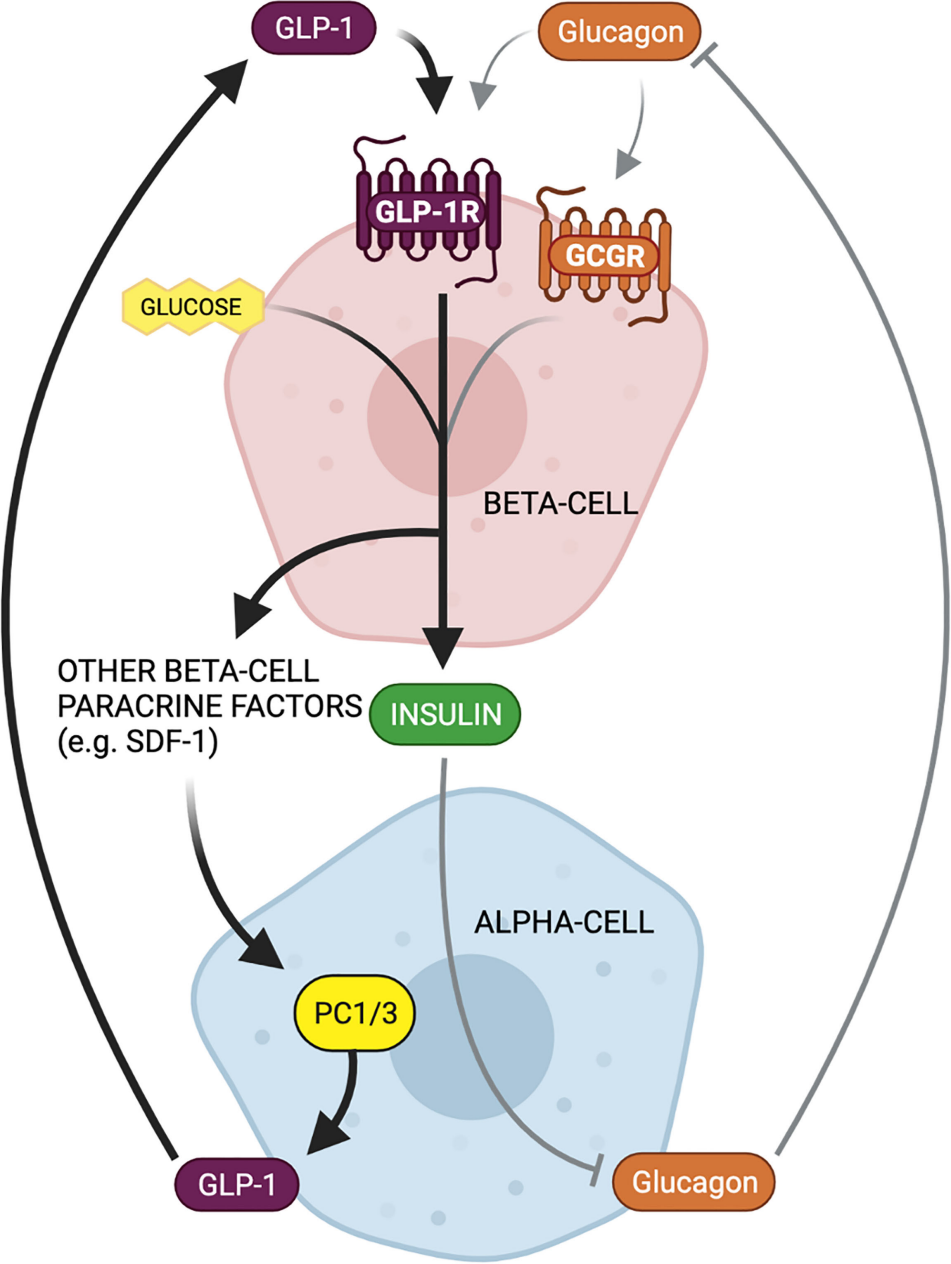
Bi-directional paracrine feedback mechanisms regulate islet function in health and disease. Alpha-cell proglucagon-derived peptides (glucagon and GLP-1) signal through the beta-cell GLP-1 receptor (GLP-1R) and glucagon receptor (GCGR) to potentiate glucose-stimulated insulin secretion. Beta-cell insulin secretion then inhibits alpha-cell glucagon secretion, thereby constituting a paracrine negative feedback loop between alpha- and beta-cells under normal physiological conditions (gray arrows). In contrast, in response to beta-cell injury or metabolic stress, there is an increased reliance on GLP-1 and beta-cell GLP-1R signaling for insulin secretion. Increased beta-cell GLP-1R signaling results in the secretion of paracrine factors that can activate alpha-cell PC1/3 expression and GLP-1 production. This locally produced GLP-1 can then favorably signal at the beta-cell GLP-1R, thereby constituting a paracrine positive feedback loop between alpha- and beta-cells as an adaptation to metabolic dysfunction.

Our group reported that enhanced beta-cell GLP-1R signaling activates alpha-cell PC1/3 expression and GLP-1 production, which is associated with improved glucose tolerance and GSIS following VSG in mice (16, 39) (**Figure 4**). Furthermore, we recently reported that pharmacological activation of beta-cell GLP-1R signaling through the administration of a GLP-1R agonist can mimic the effect of VSG to enhance alpha-cell GLP-1 production in HFD-fed mice (13). The combined interpretations from these studies support a model in which increased beta-cell GLP-1R signaling enhances alpha-cell GLP-1 production to amplify beta-cell function and insulin secretion through a local paracrine positive feedback loop of GLP-1 signaling (**Figure 5**). Moreover, our findings suggest that this paracrine feedback loop can be harnessed through the administration of GLP-1R agonists. Indeed, while enhanced GLP-1R signaling outside of the islet may mediate, at least in part, the *in vivo* effects of GLP-1R agonists on enhanced islet function, studies performed in isolated islets, in the absence of peripheral GLP-1R signaling, confirm the propensity for GLP-1R agonists to induce direct intra-islet effects (**Figure 4**). For instance, similar to our results in mice, we demonstrated that treatment of human islets with a GLP-1R agonist results in increased islet active GLP-1 production (13). Similarly, the GLP-1R agonists, liraglutide and Exendin-4, have been shown to decrease beta-cell apoptosis and improve beta-cell insulin secretory function in isolated non-diabetic (107) and T2DM (108) human islets and in rat (109) and pig (110) islets. In line with evidence that beta-cell stress induces an upregulation of intra-islet GLP-1, Tsunekawa et al. reported that the beta-cells of diabetic mice treated with Exendin-4 had decreased expression of markers of ER stress (111). Furthermore, islets from mice with alpha-cell ablation exhibit normal beta-cell secretory function in response to exogenous GLP-1 or glucagon (19), suggesting that GLP-1R agonists can augment insulin secretion independently of alpha-cell-derived proglucagon products *in vitro*. However, as an increasing body of evidence demonstrates that paracrine interactions between alpha-cells and beta-cells are essential for the coordination of insulin secretion *in vivo*, it follows, therefore, that this paracrine crosstalk is also essential for the beneficial metabolic effects of pharmaceutical agents *in vivo*. In support of this, we found that the effect of liraglutide to increase alpha-cell GLP-1 expression necessitates intact beta-cell GLP-1R signaling (13), suggesting that the efficacy of GLP-1R agonists is supported by intra-islet paracrine signaling.

In comparison to GLP-1R agonists, DPP-IV inhibitors decrease the enzymatic degradation of endogenously produced GLP-1, thereby potentiating incretin signaling pathways, albeit with lower potency than GLP-1R agonists, yet fewer adverse effects (112). *In vitro* studies performed on mouse and human islets validate that DPP-IV inhibitors can mediate direct effects on islet hormones (**Figure 4**). For example, DPP-IV inhibitors have been shown to promote beta-cell survival and function, characterized as augmented GSIS, in islets of non-diabetic and T2DM human donors (113, 114), demonstrating that ligands of DPP-IV enzymatic activity that function in a similar capacity to alpha-cell-derived proglucagon ligands are both present and active in isolated islets. Similarly, the effect of the DPP-IV inhibitors, NVPDPP728 and vildagliptin, to increase insulin secretion from isolated mouse islets was attenuated in the presence of Exendin 9-39 (115). Notably, DPP-IV targets include cytokines, growth factors, and neurotransmitters, in addition to islet-derived peptide hormones, indicating that the improvements in beta-cell function in response to DPP-IV inhibition may also be mediated by other intra-islet paracrine signaling factors. Nevertheless, Campbell et al. reported that treatment of human islets with sitagliptin resulted in a sevenfold increase in islet active GLP-1 content (14), and similarly, *in vitro* treatment of human islets with linagliptin (114) and vildagliptin (115) has been shown to stabilize active GLP-1 in islet supernatants and protect non-diabetic human islets from experimentally induced beta-cell damage (114). Finally, Hutch et al. recently reported that the glucoregulatory improvements of DPP-IV inhibitors are dependent on pancreatic proglucagon-derived factors *in vivo* (15), suggesting that these pancreatic peptides are critical substrates for the inhibitory effects of DPP-IV. Together, these results strongly suggest that islet-derived GLP-1 contributes to the effects of DPP-IV inhibitors. Moreover, these studies reveal that modulation of islet DPP-IV activity can be a valuable target for increasing islet GLP-1 activity. Indeed, Bugliani et al. recently reported that human T2D islets have lower activity of DPP-IV, as compared to non-diabetic islets (113), and furthermore, Omar et al. identified a positive correlation between DPP-IV activity in the islet and insulin secretion (115), suggesting that islets can intrinsically regulate DPP-IV to promote islet function. Therefore, these studies suggest that DPP-IV inhibitors can beneficially affect the alpha-cell to beta-cell GLP-1R signaling axis for the treatment of diabetes.

## Conclusion

In light of the recent advances in our understanding of intra-islet GLP-1 biology, alpha- to beta-cell bidirectional paracrine signaling is increasingly recognized as a key contributor to whole-body metabolic function. While the contributions of alpha-cell GLP-1 versus glucagon to the regulation of beta-cell function have yet to be established, these findings illustrate that beta-cell GLP-1R stimulation is primarily mediated by paracrine intra-islet ligands, rather than by the endocrine action of gut-derived GLP-1. Further delineation of the mechanisms underlying alpha-cell GLP-1 production is needed to fully characterize this intra-islet paracrine feedback loop. Nevertheless, these findings suggest that the regulation of alpha-cell proglucagon processing may be an unrealized target for the treatment of diabetes.

## Author contributions

Conceptualization: MH., MS, and BC. Writing—original draft preparation: MH and MS. Writing—review and editing: MH, MS, and BC. Supervision and funding acquisition: BC. All authors have read and agreed to the published version of the manuscript. All authors contributed to the article and approved the submitted version.

## Funding

This work was supported by The Hartwell Foundation and the NIH/NIDDK (R56DK124853, F30DK126538).

## Acknowledgments

Figures were created with BioRender.com.

## Conflict of interest

The authors declare that this review was prepared in the absence of any commercial or financial relationships that could be construed as a potential conflict of interest.

## Publisher’s note

All claims expressed in this article are solely those of the authors and do not necessarily represent those of their affiliated organizations, or those of the publisher, the editors and the reviewers. Any product that may be evaluated in this article, or claim that may be made by its manufacturer, is not guaranteed or endorsed by the publisher.
